# Clarifying off-target effects for torcetrapib using network pharmacology and reverse docking approach

**DOI:** 10.1186/1752-0509-6-152

**Published:** 2012-12-10

**Authors:** Shengjun Fan, Qiang Geng, Zhenyu Pan, Xin Li, Lu Tie, Yan Pan, Xuejun Li

**Affiliations:** 1 State Key Laboratory of Natural and Biomimetic Drugs, Department of Pharmacology, School of Basic Medical Sciences, Peking University and Institute of System Biomedicine, Peking University, No.38 Xueyuan Road, Beijing, 100191, China; 2 Department of Cardiology, Peking University People’s Hospital, No.11 Xizhimen South Street, Beijing, 100044, China; 3 Department of Pharmacy, Xi’an Children’s Hospital, No.69 Xijuyuan road, Xi’an, 710003, China

## Abstract

**Background:**

Torcetrapib, a cholesteryl ester transfer protein (CETP) inhibitor which raises high-density lipoprotein (HDL) cholesterol and reduces low-density lipoprotein (LDL) cholesterol level, has been documented to increase mortality and cardiac events associated with adverse effects. However, it is still unclear the underlying mechanisms of the off-target effects of torcetrapib.

**Results:**

In the present study, we developed a systems biology approach by combining a human reassembled signaling network with the publicly available microarray gene expression data to provide unique insights into the off-target adverse effects for torcetrapib. Cytoscape with three plugins including BisoGenet, NetworkAnalyzer and ClusterONE was utilized to establish a context-specific drug-gene interaction network. The DAVID functional annotation tool was applied for gene ontology (GO) analysis, while pathway enrichment analysis was clustered by ToppFun. Furthermore, potential off-targets of torcetrapib were predicted by a reverse docking approach. In general, 10503 nodes were retrieved from the integrative signaling network and 47660 inter-connected relations were obtained from the BisoGenet plugin. In addition, 388 significantly up-regulated genes were detected by Significance Analysis of Microarray (SAM) in adrenal carcinoma cells treated with torcetrapib. After constructing the human signaling network, the over-expressed microarray genes were mapped to illustrate the context-specific network. Subsequently, three conspicuous gene regulatory networks (GRNs) modules were unearthed, which contributed to the off-target effects of torcetrapib. GO analysis reflected dramatically over-represented biological processes associated with torcetrapib including activation of cell death, apoptosis and regulation of RNA metabolic process. Enriched signaling pathways uncovered that IL-2 Receptor Beta Chain in T cell Activation, Platelet-Derived Growth Factor Receptor (PDGFR) beta signaling pathway, IL2-mediated signaling events, ErbB signaling pathway and signaling events mediated by Hepatocyte Growth Factor Receptor (HGFR, c-Met) might play decisive characters in the adverse cardiovascular effects associated with torcetrapib. Finally, a reverse docking algorithm *in silico* between torcetrapib and transmembrane receptors was conducted to identify the potential off-targets. This screening was carried out based on the enriched signaling network analysis.

**Conclusions:**

Our study provided unique insights into the biological processes of torcetrapib-associated off-target adverse effects in a systems biology visual angle. In particular, we highlighted the importance of PDGFR, HGFR, IL-2 Receptor and ErbB1tyrosine kinase might be direct off-targets, which were highly related to the unfavorable adverse effects of torcetrapib and worthy of further experimental validation.

## Background

Cardiovascular disease remains to be the most unexceptional cause of morbidity over the past few years in spite of the usage of hydroxymethylglutaryl coenzyme A (HMG CoA) reductase inhibitors (statins) that lower low-density lipoprotein (LDL) cholesterol [1]. Elevated LDL or lowered high-density lipoprotein (HDL) cholesterol level is a crucial risk factor for cardiovascular ailments [2,3]. Accordingly, raising HDL induced by cholesteryl ester transfer protein (CETP) inhibition is an attractive tactic for anti-atherosclerosis, which may reduce the residual risk of cardiovascular events [4].

**Figure 1:**
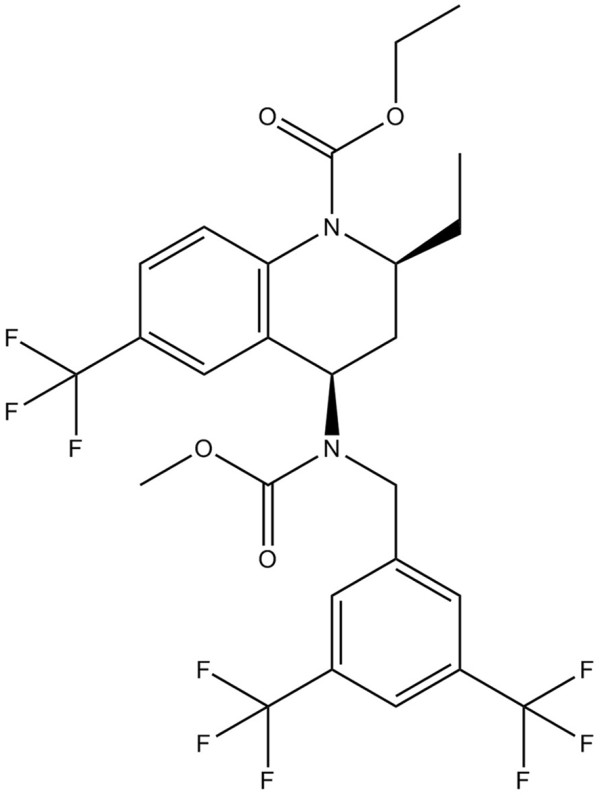
**Chemical structure of torcetrapib.** ChemSpider (http://www.chemspider.com/) ID: 140123; Molecular Formula: C_26_H_25_F_9_N_2_O_4_; Average mass: 600.473328 Da; Systematic name: Ethyl(2R,4S)-4-{[3,5-bis(trifluoromethyl)benzyl](methoxycarbonyl)amino}-2-ethyl-6-(trifluoromethyl)-3,4-dihydro-1(2H)-quinolinecarboxylate.

Torcetrapib (Figure 1), a CETP inhibitor firstly proposed by Pfizer Inc., had been characterized to suppress the exchange of HDL and triglyceride-rich lipoprotein in patients with hyperlipidemia, which resulted in the elevation of HDL in the peripheral circulatory system [5]. However, torcetrapib was found to be associated with incremental mortality and cardiovascular event risk, including activated aldosterone system and induced hypertension in the ILLUMINATE trial [6]. Off-target effects occurred via inhibition of a kinase not intended to be targets for drugs. So far, the detailed mechanisms underlying the off-target adverse effects of torcetrapib are quite limited and remain obscure.

With the rapid development of high-throughput screen (HTS) technology such as microarray, the superiority of systems biology and network pharmacology gradually embodied [7,8]. Reconstructing networks of biological organism through integrating diverse sources are crucial for comprehending biological processes associated with pathema. Computational biology provides profitable patronage to address the scientific suspense through pragmatic modeling and theoretical exploration, which furnish a brand-new network poly-pharmacology approach for drug identification and discovery [9]. Based on systems biology, it affords a rewarding assistance to improve drug potency and forecast the unwanted off-target effects at a higher efficiency and lower attrition, especially for a new generation of known drugs [10]. In addition, as a crucial technology in drug discovery, reverse docking approach also revealed a prominent performance in understanding the basis of a drug and receptors which provided benignant avails in drug target identification [11].

To better expound the unfavorable adverse reactions of torcetrapib, a novel network systems approach was proposed by integrating high quality manually curated data with microarray gene expression profiling into a context-specific network, which allowed us to explicate the off-target adverse effects of torcetrapib in a different angle. Detailed illustrations are as follows.

## Results and discussion

Although statins had been well characterized as the best studied contemporary cardiovascular therapies over the past few years, the optimal approach to LDL reduction remained to be controversial. Meanwhile, the prejudice of low levels of HDL cholesterol in cardiovascular system became increasingly prominent, which had a tight consanguinity with myocardial infarction and death from coronary heart disease (CHD). Thus, strategies targeting HDL had been a therapeutic tactic for anti-atherosclerosis. As a novel CETP inhibitor, torcetrapib had been recognized as one of the auspicious foremost candidates for elevating HDL. However, owing to its high risk of mortality, torcetrapib experienced the battle of “Waterloo”, which overshadowed the entire prospect of anti-cholesterol drugs.

With the speedy development of bioinformatics, organization of knowledge on drug, disease and target inaugurated a brand-new era in drug target identification and discovery. Network pharmacology comprehended the complexity of biological processes by integrating network biology and poly-pharmacological perspective to create predictive models [12]. Network reconstruction and integration of aberrant genes involved in drugs could uncover the capital gene regulatory networks (GRNs) modules which led to the dysfunction of regular biological systems.

After integrating HPRD (Human Protein Reference Database, http://www.hprd.org/) with a manually curated human signaling network acquired from Cui et al. [13], the over-expressed microarray data originated from human adrenal carcinoma cells treated with torcetrapib were mapped to construct the context-specific network. Cytoscape (http://www.cytoscape.org/), an open source package for visualizing complex networks and integrating diverse types of resources, is an indispensable platform for bioinformatics, social network analysis and network pharmacology [14]. The drug-gene interaction network of torcetrapib was established utilizing three plugins, including BisoGenet [15], NetworkAnalyzer and ClusterONE [16]. Molecular relations (protein-protein and protein/DNA interactions) were connected based on SysBiomics platform (http://biomine.cigb.edu.cu/sysbiomics/). GRNs communities, which reflected the situation of torcetrapib-associated over-expressed genes, were detected in MCODE algorithm. The DAVID functional annotation tool (http://david.abcc.ncifcrf.gov/) [17,18] and ToppFun web server (http://toppgene.cchmc.org/enrichment.jsp) [19] were employed freely to identify the significantly-represented biological processes and the enriched signaling pathways, respectively.

An *in silico* drug target reverse searching method was applied for screening potential off-targets of torcetrapib. Reverse docking, a flexible ligand-receptors inverse docking program, conducted computer-automated search of potential targets of a small molecule by docking it to a cavity of each receptor. To optimize docking parameter, an accurate docking module in Discovery Studio (version 2.5, Accelrys) named CDOCKER was employed. The cavity of each protein was derived from the three dimensional structures of Protein Data Bank (PDB, http://www.rcsb.org/) based on the enriched pathways. Proteins with high binding affinity with torcetrapib were considered to be the most potential direct off-targets.

### Torcetrapib-associated signaling map construction

**Figure 2:**
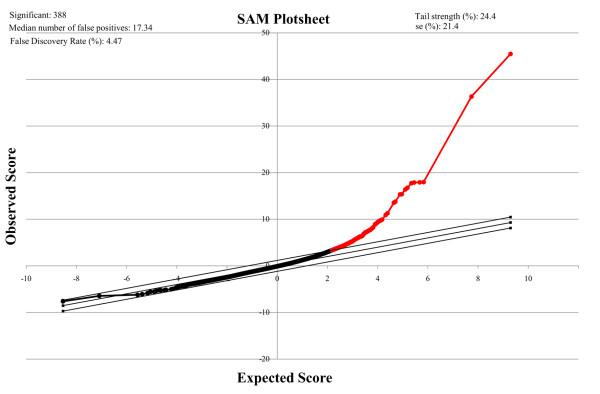
**SAM plot sheet output of the gene expression profiling of the microarray dataset from a study of torcetrapib (GEO: GDS3556).** SAM plot sheet illustrated a signature for differentially expressed genes of H295 adrenal carcinoma cells treated with torcetrapib. Red dots represented gene sets up-regulated.

Totally, 388 differentially expressed genes were identified by SAM (Additional file 1). As shown in Figure 2, with the assistance of SAM Plot Controller, we draw a band of two parallel lines with a distance of 1.1724 (delta value) according to the False Discovery Rate (FDR) threshold of 0.05. After combining HPRD (Raw data is available in Additional file 2) with a manually curated human signaling network obtained from Cui et al. [13], an integrated human signaling network contained 10503 nodes and 47660 edges were connected on the basis of SysBiomics platform, which amassed miscellaneous data from BIND, HPRD, MINT, DPI, BIOGRID and INTACT [15]. To uncover torcetrapib-associated regulatory network, 215 out of the 388 significantly up-regulated genes were mapped to illustrate the context-specific network.

### GRNs modules excavation

**Figure 3:**
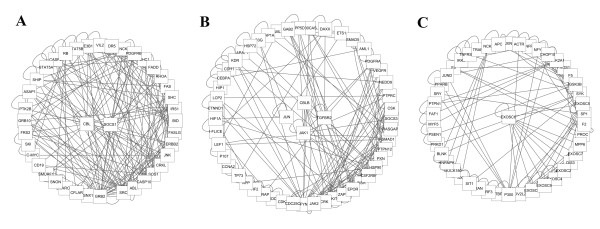
**Significant up-regulated torcetrapib signature driving genes (central location) in the gene regulatory networks (GRNs) modules (circle layout).** (**A**) For GRN1. (**B**) For GRN2. (**C**) For GRN3.

Genes in biological networks always enjoy a similarity in which they are more intimately connected to implement particular biological functions. This kind of dense clique-like structure within a network theme is termed as GRNs modules or gene sets [20]. GRNs, the specific sub-networks that gave rise to the dysfunction of regulator in biological systems, were critical in maintaining the stability of the entire network. Thus, analysis of gene lists regulated by the over-represented microarray genes was propitious to annotate the specific biological processes involved in torcetrapib-associated undesired off-target effects. Currently, we utilized the MCODE algorithm in ClusterONE plugin, which searched nodes for expansion by computing a score of local density for each node in a graph, to detect the dominant controller of gene regulation associated with torcetrapib. Totally, the largest three principal modules (Figure 3) encoded by torcetrapib-gene expression profiling (with score above 2.0) were excavated and the complete lists of the core GRNs were presented in Additional file 3.

### Gene ontology (GO) analysis

**Figure 4:**
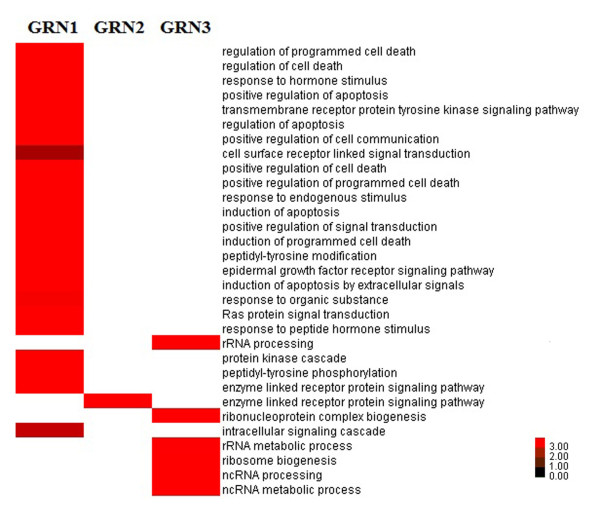
**Heatmap of the over-represented biological processes associated with torcetrapib generated by Cluster/TreeView.** Columns represented significant over-represented biological processes, while rows corresponded to gene regulatory networks (GRNs) modules. Expression values were logarithm of ratio value utilizing log transform data. Red color in each grid represented positive, while white represented null.

To assess the capital GRNs in biological processes, the DAVID functional annotation tool was utilized [17,18]. Our results for the enriched over-represented biological processes implicated in torcetrapib were presented in Figure 4 (FDR<0.01). Of note, most of these functions were highly bound up with cell death, apoptosis, signaling transduction, tyrosine modification and regulation of RNA metabolic process.

### Pathway enrichment analysis

Pathway, a set of genes that acted together to implement certain biological functions, was an excellent indicator to annotate dysregulation in view of gene regulation. Thus, we speculated the regulated pathways of the momentous gene sets based on ToppFun, a web server for comparative enrichment analysis of multiple gene lists [19]. Significant over-expressed pathways of the capital GRNs were listed in Table 1 (FDR<0.05). Accordingly, torcetrapib mainly influenced IL-2 Receptor Beta Chain in T cell Activation, Platelet-Derived Growth Factor Receptor (PDGFR) beta signaling pathway, IL2-mediated signaling events, ErbB signaling pathway and signaling events mediated by Hepatocyte Growth Factor Receptor (HGFR, c-Met) via up-regulation of CBL, SOCS1, JAK1, JUN, TGFBR2 and EXOSC6.

**Table 1:** Main enriched signaling pathways of torcetrapib related to its adverse reactions (FDR<0.05)

**Index**	**Driving genes**	**Pathways**	***p*-value**
GRN1	CBL,SOCS1	BioCarta: IL-2 Receptor Beta Chain in T cell Activation	4.112E-18
	CBL,SOCS1	NCI-Nature Curated: PDGFR-beta signaling pathway	5.309E-12
	SOCS1	NCI-Nature Curated: IL2-mediated signaling events	1.491E-10
	CBL	KEGG pathway: ErbB signaling pathway	1.838E-10
GRN2	JUN	NCI-Nature Curated: Signaling events mediated by Hepatocyte Growth Factor Receptor (c-Met)	1.175E-4
GRN3	EXOSC6	Reactome: Genes involved in mRNA Decay by 3’ to 5’ Exoribonuclease	4.537E-14
	EXOSC6	Reactome: Genes involved in Metabolism of mRNA	3.187E-8
	EXOSC6	Reactome: Genes involved in Metabolism of RNA	6.834E-6

*FDR*: false discovery rate.

### Reverse docking analysis

Predicting potential binding receptors of ligands by docking protocol could assist in new targets discovery and identification. Reverse docking approach, the opposite of the direct docking method firstly proposed by Chen et al. [11], could identify probable binding proteins for a specific small molecule. CDOCKER, an accurate docking module in Discovery Studio, is a powerful tool to predict the conformation and related binding energies of ligand-receptor complexes. In the present study, performance was conducted by docking torcetrapib to a series of proteins based on the enriched signaling pathways. Our results for reverse docking targets of torcetrapib were listed in Table 2.

**Table 2:** Off-targets candidates for torcetrapib identified by reverse docking procedure

**Rank**	**Target details (PDB Code)**	**Ligand**	**Binding score (kcal/mol)**
1	PDGFR (1GQ5)	crenolanib	28.7711
		torcetrapib	42.0439
2	HGFR (3U6H)	compound 03X	40.8298
		torcetrapib	40.2422
3	IL-2 receptor (4HCV)	compound 13 J	46.049
		torcetrapib	38.126
4	ErbB1 (3BEL)	compound POX	35.5674
		torcetrapib	34.7466

### IL2-mediated signaling events and activation of T cell receptor pathway mediated by IL-2 gave rise to the unwanted effects for torcetrapib

Among the myriad of intra-cellular signaling networks that governed the pathogenesis of cardiovascular event, activation of T cell receptor signaling mediated by IL-2 awoke our concern. Recently, numerous evidences illustrated that the pathological proceeding of atherosclerosis had an intimate relation with chronic inflammation [21]. As a primary regulator of immune cell, the characteristics of T cell receptor pathway mediated by IL-2 in atherosclerosis had been certificated [22-25]. Lipid deposition and infiltration of inflammatory cells were responsible for the formation of atherosclerosis and a variety of cells such as T lymphocytes, monocytes, macrophages, endothelial cells, platelet and vascular smooth muscle cells were engaged in the occurrence and progression of atherosclerosis. Meanwhile, leukocyte adhesion molecules and inflammatory chemokines were other elements which facilitated the accumulation of plaques. T cells activated by IL-2 in the arterial vessel played a momentous function in atherosclerosis, which induced apoptosis of vascular smooth muscle cells and facilitated the formation of plaques [26].

Similarly, hypertension is also considered to be an inflammatory pathema [27,28]. Considerable documents illustrated that T cells could stimulate the release of cytokines and inflammatory factors, which resulted in hypertension and myocardial fibrosis. As a vasoactive peptide, angiotensin II (AngII) was identified as a crucial factor in the development of hypertension. Activated T cells mediated by IL-2 had been authenticated to be rich in AngII receptor, which could promote the migration of dendritic cells [29] and amplify inflammation through autocrine [30,31]. More and more evidences attested the relations between experimental hypertension and T cell immune activation. Guzik et al. [32] found that mice continuously infiltrated with AngII exhibited extraordinary abnormalities of T cell. Further studies disclosed that AngII significantly increased the amount of T cell in the perivascular adipose tissue via enrichment of CD69/CD44 or activation of Chemokines (C-C motif) receptor 5, which subsequently elevated the level of T lymphocytes in the peripheral circulatory system. Thus, the off-target prediction was applied by docking torcetrapib to IL-2 receptor.

**Figure 5:**
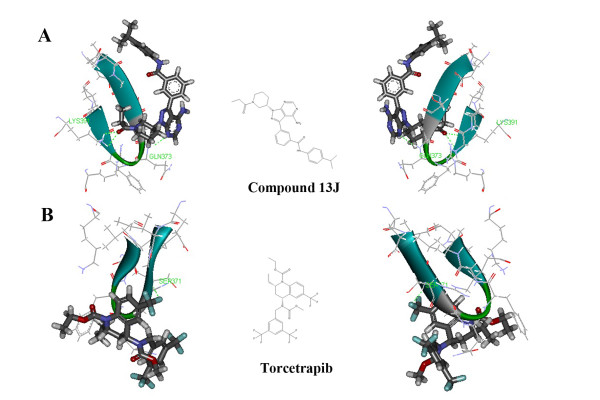
**Prediction of IL-2 receptor as a possible off-target of torcetrapib.** (**A**) Docked complex of IL-2 receptor (PDB code 4HCV) and compound 13 J in the best docking pose. (**B**) Docked complex of IL-2 receptor and torcetrapib in the best docking pose. Compound 13 J and torcetrapib were in the stick representation, whilst the amino acid residues of IL-2 receptor were displayed by solid ribbon style in the line representation. C, H, O, N and F were colored with gray, white, red, blue and brown, respectively.

The X-ray crystallography of IL-2 receptor with an endogenous ligand (compound 13 J, 3-{4-amino-1-[(3S)-1-propanoylpiperidin-3-yl]-1H-pyrazolo[3,4-d]pyrimidin-3-yl}-N-[4- (propan-2-yl)phenyl]benzamide) was downloaded from PDB (PDB code 4HCV). Compound 13 J (Figure 5A), a nonreceptor tyrosine kinase Itk (interleukin-2 inducible T- cell kinase) blocker, exhibited positive activities with IC_50_ 0.4 μM. Figure 5 showed the results of the calculations. The docking protocol revealed that both compound 13 J and torcetrapib could cage into the IL-2 receptor binding pocket. The interaction energy of torcetrapib and IL-2 receptor complex was decreased compared with compound 13 J (compound 13 J, 46.049 kcal/mol; torcetrapib, 38.126 kcal/mol). Docking consequences elaborated that the conserves amino acid residues LYS391, GLN373 and SER371 in IL-2 receptor played a decisive role in maintaining the functional conformation and directly involved in compound 13 J and torcetrapib binding.

### PDGFR-beta signaling pathway and the adverse effects of torcetrapib

Platelet derived growth factor (PDGF), a 24ku cationic glycoprotein, mainly indwelt in platelet alpha granule, impaired endothelial cell, macrophages, smooth muscle cells, fibroblasts and mesangia cells, which mediated multiple interactions between tissues and endothelial cells through releasing PDGF in an autocrine and paracrine chain amplificated reaction forms [33,34]. A variety of mechanisms involved in the development of atherosclerosis had been reported to be highly associated with PDGF. Cagnin et al. [35] discovered that a high level of PDGF and interleukin was detected in patients with atherosclerosis, suggesting that PDGF could influence the proceeding of atherosclerosis in association with inflammatory factors. Additionally, Cha et al. [36] also observed proliferation and migration in smooth muscle cell after PDGF treatment in cultured human aortic smooth muscle cells *in vitro*, which indicated that PDGF could facilitate the formation of atherosclerosis via accelerating the migration and proliferation of plaque.

Despite the fact that percutaneous coronary intervention (PCI) was one of the most effective therapeutic approaches for CHD by far, restenosis after stenting was still unavoidable, which affected the long term efficacy. Li and colleagues [37] disclosed that the increased expression of PDGF mRNA was found on carotid artery balloon dilatation rat. Experimental results suggested that PDGF could activate its upstream pathways via directly binding PDGFR-β, which initiated intermediate signal protein, activated mitogen activated protein kinase pathway (MAPK) cascade afterwards and promoted proliferation, migration and angiogenesis in smooth muscle cells through dimerization and autophosphorylation of tyrosine residues phosphorylated [38,39]. Chintalgattu’s research [40] uncovered an elevation of PDGFR-β in cardiac pressure overload mice, implicating that PDGFR-β was a compensatory reaction in heart under pressure load, which depicted the intimate relationship between the activation of PDGFR signaling pathway and cardiovascular diseases.

**Figure 6:**
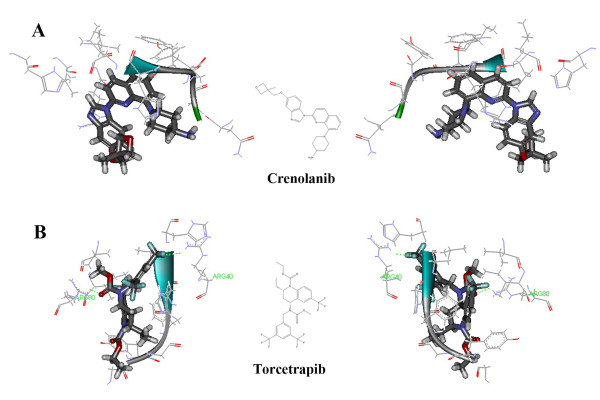
**Prediction of PDGFR as a possible off-target of torcetrapib.** (**A**) Docked complex of PDGFR (PDB code 1GQ5) and crenolanib in the best docking pose. (**B**) Docked complex of PDGFR and torcetrapib in the best docking pose. Crenolanib and torcetrapib were in the stick representation, whilst the amino acid residues of PDGFR were displayed by solid ribbon style in the line representation. C, H, O, N and F were colored with gray, white, red, blue and brown, respectively.

Owing to the availability of synthetical PDGF tyrosine kinase inhibitors, it might be conceivable to use crenolanib to exploit the binding pocket region of PDGF protein. Figure 6 illustrated that torcetrapib perfectly matched the crystallographic position of the PDGF tyrosine kinase receptor (PDB Code 1GQ5) with 42.0439 kcal/mol by directly interacting with ARG40 and ARG80 in its besting docking pose. As is well known, H-bonds play a vital role in the stability of structure and function of biological molecules. The presence of interaction between ARG40 and ARG80 is interesting, which had been identified as the most important amino acid residue in the formation of hydrogen bond.

### Aberrant ErbB pathway was associated with the off-target effects for torcetrapib

Except for cancer, the ErbB family of four receptor tyrosine kinases (ErbB1, ErbB2, ErbB3 and ErbB4) also engaged in certain non-neoplastic pathologies, such as hypertension [41], infectious diseases [42] and chronic renal dysfunction [43]. More recent studies have demonstrated that neuregulins (NRGs)/ErbB1 signaling pathway was essential for normal myocardial development and pathological vasoconstriction, especially in cardiac smooth muscle [44]. One such momentous NRGs was heparin-binding (HB)-EGF. Hao et al. [41] reported that the activation of ErbB1 receptor mediated by HB-EGF played a significant role in cardio-vasculature and hypertension, which facilitated the formation of atherosclerotic plaque and vascular stenosis.

**Figure 7:**
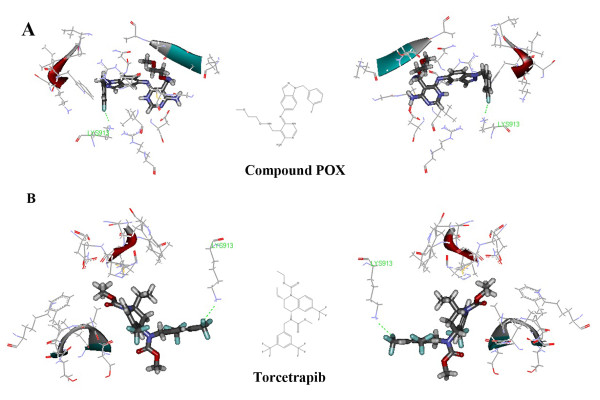
**Prediction of ErbB1 tyrosine kinase as a possible off-target of torcetrapib.** (**A**) Docked complex of ErbB1 (PDB code 3BEL) and compound POX in the best docking pose. (**B**) Docked complex of ErbB1 and torcetrapib in the best docking pose. Compound POX and torcetrapib were in the stick representation, whilst the amino acid residues of ErbB1 tyrosine kinase were displayed by solid ribbon style in the line representation. C, H, O, N and F were colored with gray, white, red, blue and brown, respectively.

The three dimensional structure of ErbB1 tyrosine kinase with compound POX (4-amino-6-arylaminopyrimidine-5-carbaldehyde oximes) was downloaded from PDB (code number 3BEL). As shown in Figure 7, the binding energy between torcetrapib and ErbB1 tyrosine kinase experienced a lower reduction in the best docking pose compared with compound POX (POX, 35.5674 kcal/mol; torcetrapib, 34.7466 kcal/mol). Of note, the presence of interaction in LYS913 aroused our curiosity, which was proposed as the most prominent amino acid residue in the stability of ErbB1 and POX/torcetrapib.

### HGFR pathway contributed to the unfavorable effects of torcetrapib

As a heparin binding glycoprotein originated from mesenchymal cells, hepatocyte growth factor (HGF) possessed various biological activities including regulating mitosis, morphogenesis, hematopoiesis, myocardial hypertrophy, angiogenesis, fibrosis and tissue regeneration, which were took effect via binding HGF specific receptor kinase (c-Met) [45]. HGF promoted mitosis and revealed anti-apoptosis effect on vascular endothelial cells. Meanwhile, there was no stimulation of HGF on the growth of smooth muscle cells, suggesting that it was a specific endothelial cell growth factor and injury repaired factor [46]. Previously, we found that HGF played profitable prothetic roles in the pathogenesis of CHD, especially for atherosclerosis. The autocrine or paracrine mechanisms of HGF was reduced by high concentration of transforming growth factor β (TGF-β) and AngII after endothelial damage in atherosclerosis, which resulted in the elevation of serum HGF produced by lung, liver and kidney to regulate the proliferation or migration of vascular endothelial and smooth muscle cells [47,48].

**Figure 8:**
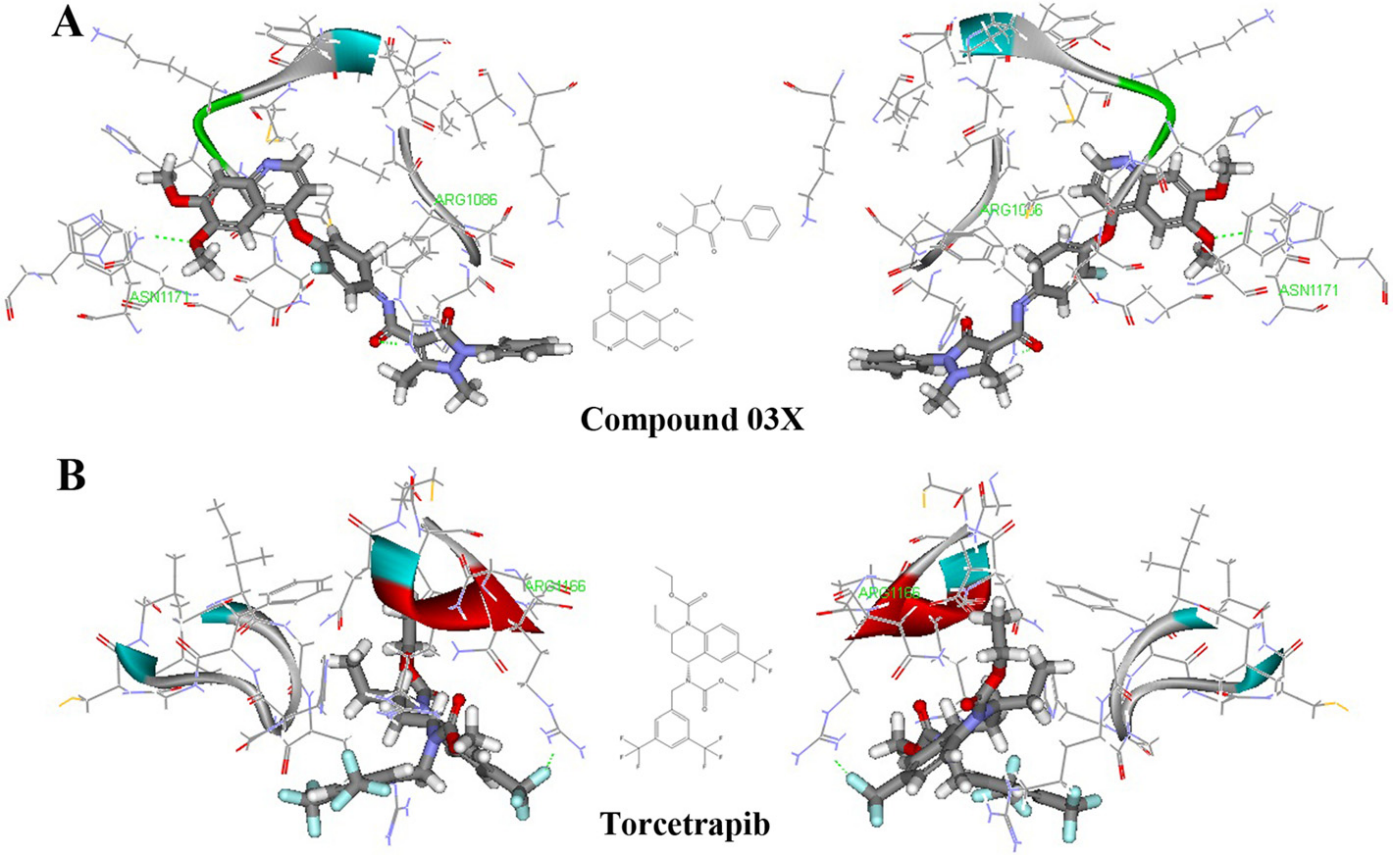
**Prediction of HGFR as a possible off-target of torcetrapib.** (**A**) Docked complex of HGFR (PDB code 3UH6) and compound 03X in the best docking pose. (**B**) Docked complex of HGFR and torcetrapib in the best docking pose. Compound 03X and torcetrapib were in the stick representation, whilst the amino acid residues of HGFR were displayed by solid ribbon style in the line representation. C, H, O, N and F were colored with gray, white, red, blue and brown, respectively.

The three dimensional crystal structure of HGF receptor and compound 03X (N-{4-[(6,7-dimethoxyquinolin-4-yl)oxy]-3-fluorophenyl}-1,5-dimethyl-3-oxo-2-phenyl-2,3-dihydro-1H-pyrazole-4-carboxamide) showed that the 6-dimethoxyquinoline and the carbonyl group in 1,5-Dimethyl-3-oxo-2-phenyl-2,3-dihydro-1H-pyrazole-4-carboxamide could interact with the hinge region of the active site via directly coalescing with ARG1086 and ASN1171 (Figure 8). Similarly, a H-bond (ARG1166) adjacent to the 6-(trifluoromethyl)-1, 2, 3, 4-tetrahydroquinoline ring also contributed to the stability of torcetrapib and HGF receptor.

As a CETP inhibitor, torcetrapib could activate relevant signaling pathways mentioned above through directly binding PDGFR, HGFR, IL-2 Receptor and ErbB1tyrosine kinase and up-regulating CBL, SOCS1, JAK1, JUN, TGFBR2 and EXOSC6 afterward, which subsequently exerted the exacerbation of endothelium injury and increased cardiovascular events [6]. Thus, a synergetic combination of anti-hypertensive drugs such as angiotensin converting enzyme inhibitors (ACEIs) was proposed to be an effective and beneficial strategy to decrease torcetrapib-associated off-target unfavorable effects in cardiovascular system [49].

## Conclusions

A whole genomic drug-gene interaction network based on the integrative manually curated signaling network and microarray profiles was established to explicate the potential off-target effects for torcetrapib. Totally, three momentous GRNs modules which might have a close relationship with the unwanted effects of torcetrapib were mined. Meanwhile, enriched analysis was carried out and certain significant enriched pathways were detected, which had been reported to have a definite correlation with cardiovascular maladjustment. In particular, we highlighted the importance of IL-2 Receptor Beta Chain in T cell Activation, PDGFR-beta signaling pathway, IL2-mediated signaling events, ErbB signaling pathway and signaling events mediated by HGFR (c-Met) and revealed that PDGFR, HGFR, IL-2 Receptor and ErbB1tyrosine kinase were direct off-targets for torcetrapib.

Taken together, these findings suggested that the network off-target effects prediction methods *in silico* were profitable for illustrating the relationship between drug and disease related off-targets for interventions. However, due to the false positive connection and noises in the reassembled network, the predictive model in this study was still far more completed. We proposed that our study on the off-target effects of torcetrapib based on network pharmacology will provide beneficial insights for further experimental validations.

## Methods

### Microarray data analysis

The microarray gene expression profiling associated with torcetrapib was acquired from the National Center for Biotechnology Information (NCBI) Gene Expression Omnibus (GEO, http://www.ncbi.nlm.nih.gov/geo/) database under the accession number GDS3556 [50]. This data set was derived from a study on H295 adrenal carcinoma cells treated with blank solvent, AngII and torcetrapib. Analysis of differently expression gene was performed by Significance Analysis of Microarray (SAM) [51]. If the fold change>1.2 and False Discovery Rate (FDR)<0.05, gene expression was considered significantly different.

### Human signaling network construction

To establish a comprehensive human signaling network, we manually curated the cellular signaling molecules which integrated diverse pathways resources including BioCarta, literature-mined network, Cancer Cell Map [13] and HPRD. An open source platform for complex network analysis and visualization named Cytoscape was freely utilized to assemble the drug-gene interaction network [14]. Molecular inter-relations in the integrative network were added using BisoGenet plugin from various databases including BIND, HPRD, MINT, DPI, BIOGRID and INTACT [15].

### Functional enrichment analysis

Functional enrichment analysis was applied to identify primary biological processes, which provided clues to the underlying molecular mechanisms related to the adverse effects of torcetrapib. Significant clustering of genes was mined by MCODE algorithm [16]. All GRNs modules were classified by DAVID functional annotation tool [17,18] to perform GO analysis on the basis of “GOTERM_BP_FAT”, whilst pathway enrichment analysis was clustered by ToppFun [19].

### Ligand preparation

Chemical structures of all ligands utilized in reverse docking protocol were generated by CambridgeSoft ChemOffice 2008. Ligands were prepared by adding charges, hydrogen and applying force field in Discovery Studio environment. Energy was also minimized with ChARMm force field before performing docking. The random conformations search of torcetrapib was conducted utilizing a high temperature simulated annealing dynamics scheme. Ligands were heated to 700 K in 2000 steps, followed by annealing to 300 K in 5000 steps. Ten random conformations were generated and a final minimization was introduced to each docking poses.

### Receptor preparation

The three dimensional structures of proteins were obtained from PDB, which contains information about experimentally-determined structures of proteins, nucleic acids and complex assemblies. Drug targets were downloaded with high resolution and without mutation or missing residues around the active site. Ligands, oligomeric chains, water molecules or solvent were spilt from proteins. All proteins were remedied through the “Prepare Protein” command in Discovery Studio protocols, which added hydrogen, fixed the missing side chains, corrected connectivity or bond orders and adjusted residue protonation states to PH 7.0.

### Binding site analysis

For binding site identification, a ligand-based approach was used for identifying the potential binding sites via “Define and Edit Binding Site” tool in Discovery Studio. Ligand-based similarity search method, a strategy utilizing compounds that are known to bind to the desired targets to identify the targets of other compounds with similar properties, is an indispensable technology that is gaining increasing usage in drug discovery. In the present study, search was performed on the global surface of the protein by similarity and substructure searching [52], and the automatic identification of binding sphere was considered as highly significant.

### Targets prediction

A reverse docking algorithm, the opposite of a “direct” docking approach, was conducted by CDOCKER to hunt for potential targets of torcetrapib based on the enriched signaling pathways. CDOCKER, an implementation protocol in Discovery Studio environment, is a grid-based simulated annealing (several cycles) docking method through CHARMm force field docking tool [53]. Docking was performed using the default setting, which can avoid a potential reduction in docking accuracy.

## Competing interests

The authors declare that they have no competing interests.

## Authors’ contributions

XJL and SJF conceived this study. XJL, SJF, QG and ZYP carried out the data analysis, simulations, drafted the manuscript and analyzed the results. By carried out extensive revisions to the manuscript. All authors read and approved the final manuscript.

## Supplementary Material

Additional file 1 List of significant over-expressed genes by Significance Analysis of Microarray (SAM). Click here for file

Additional file 2 List of raw genes obtained from Human Protein Reference Database (HPRD). Click here for file

Additional file 3 List of the core gene regulatory networks (GRNs) up-regulated by torcetrapib. Click here for file

## References

[B1] Bays H Stein EA Pharmacotherapy for dyslipidaemia–current therapies and future agents Expert Opin Pharmacother 2003 4 11 1901 1938 10.1517/14656566.4.11.1901 14596646

[B2] Assmann G Gotto AJ HDL cholesterol and protective factors in atherosclerosis Circulation 2004 109 23 Suppl 1 I8 I14 10.1161/01.CIR.0000131512.50667.46 15198960

[B3] Demarin V Lisak M Morovic S Cengic T Low high-density lipoprotein cholesterol as the possible risk factor for stroke Acta Clin Croat 2010 49 4 429 439 21830454

[B4] Shinkai H Cholesteryl ester transfer-protein modulator and inhibitors and their potential for the treatment of cardiovascular diseases Vasc Health Risk Manag 2012 8 323 331 22661899 10.2147/VHRM.S25238 PMC3363149

[B5] Clark RW Sutfin TA Ruggeri RB Willauer AT Sugarman ED Magnus-Aryitey G Cosgrove PG Sand TM Wester RT Williams JA Perlman ME Bamberger MJ Raising high-density lipoprotein in humans through inhibition of cholesteryl ester transfer protein: an initial multidose study of torcetrapib Arterioscler Thromb Vasc Biol 2004 24 3 490 497 10.1161/01.ATV.0000118278.21719.17 14739125

[B6] Barter PJ Rye KA Tardif JC Waters DD Boekholdt SM Breazna A Kastelein JJ Effect of torcetrapib on glucose, insulin, and hemoglobin A1c in subjects in the investigation of lipid level management to understand its impact in atherosclerotic events (ILLUMINATE) trial Circulation 2011 124 5 555 562 10.1161/CIRCULATIONAHA.111.018259 21804130

[B7] Hopkins AL Network pharmacology: the next paradigm in drug discovery Nat Chem Biol 2008 4 11 682 690 10.1038/nchembio.118 18936753

[B8] Kitano H Systems biology: a brief overview Science 2002 295 5560 1662 1664 10.1126/science.1069492 11872829

[B9] Kortagere S Lill M Kerrigan J Role of computational methods in pharmaceutical sciences Methods Mol Biol 2012 929 21 48 10.1007/978-1-62703-050-2_3 23007425

[B10] Xie L Xie L Bourne PE Structure-based systems biology for analyzing off-target binding Curr Opin Struct Biol 2011 21 2 189 199 10.1016/j.sbi.2011.01.004 21292475 PMC3070778

[B11] Chen YZ Zhi DG Ligand-protein inverse docking and its potential use in the computer search of protein targets of a small molecule Proteins 2001 43 2 217 226 10.1002/1097-0134(20010501)43:2<217::AID-PROT1032>3.0.CO;2-G 11276090

[B12] Zhao S Iyengar R Systems pharmacology: network analysis to identify multiscale mechanisms of drug action Annu Rev Pharmacol Toxicol 2012 52 505 521 10.1146/annurev-pharmtox-010611-134520 22235860 PMC3619403

[B13] Cui Q Ma Y Jaramillo M Bari H Awan A Yang S Zhang S Liu L Lu M O’Connor-McCourt M Purisima EO Wang E A map of human cancer signaling Mol Syst Biol 2007 3 152 18091723 10.1038/msb4100200 PMC2174632

[B14] Shannon P Markiel A Ozier O Baliga NS Wang JT Ramage D Amin N Schwikowski B Ideker T Cytoscape: a software environment for integrated models of biomolecular interaction networks Genome Res 2003 13 11 2498 2504 10.1101/gr.1239303 14597658 PMC403769

[B15] Martin A Ochagavia ME Rabasa LC Miranda J Fernandez-de-Cossio J Bringas R BisoGenet: a new tool for gene network building, visualization and analysis BMC Bioinforma 2010 11 91 10.1186/1471-2105-11-91 PMC3098113 20163717

[B16] Nepusz T Yu H Paccanaro A Detecting overlapping protein complexes in protein-protein interaction networks Nat Methods 2012 9 5 471 472 10.1038/nmeth.1938 22426491 PMC3543700

[B17] Huang DW Sherman BT Lempicki RA Systematic and integrative analysis of large gene lists using DAVID bioinformatics resources Nat Protoc 2009 4 1 44 57 19131956 10.1038/nprot.2008.211

[B18] Huang DW Sherman BT Lempicki RA Bioinformatics enrichment tools: paths toward the comprehensive functional analysis of large gene lists Nucleic Acids Res 2009 37 1 1 13 10.1093/nar/gkn923 19033363 PMC2615629

[B19] Chen J Bardes EE Aronow BJ Jegga AG ToppGene Suite for gene list enrichment analysis and candidate gene prioritization Nucleic Acids Res 2009 37 Web Server issue W305 W311 19465376 10.1093/nar/gkp427 PMC2703978

[B20] Davidson E Levin M Gene regulatory networks Proc Natl Acad Sci U S A 2005 102 14 4935 10.1073/pnas.0502024102 15809445 PMC556010

[B21] Tousoulis D Davies G Stefanadis C Toutouzas P Ambrose JA Inflammatory and thrombotic mechanisms in coronary atherosclerosis Heart 2003 89 9 993 997 10.1136/heart.89.9.993 12923007 PMC1767836

[B22] Mottaghi A Salehi E Sezavar H Keshavarz SA Eshraghian MR Rezaei N Rejali L Saboor-Yaraghi AA The in vitro effect of oxidized LDL and PHA on proliferation and gene expression of regulatory T cells in patients with atherosclerosis Iran J Allergy Asthma Immunol 2012 11 3 217 223 22947906

[B23] Dinh TN Kyaw TS Kanellakis P To K Tipping P Toh BH Bobik A Agrotis A Cytokine therapy with interleukin-2/anti-interleukin-2 monoclonal antibody complexes expands CD4 + CD25 + Foxp3+ regulatory T cells and attenuates development and progression of atherosclerosis Circulation 2012 126 10 1256 1266 10.1161/CIRCULATIONAHA.112.099044 22851544

[B24] Xiong YS Wu AL Lin QS Yu J Li C Zhu L Zhong RQ Contribution of monocytes Siglec-1 in stimulating T cells proliferation and activation in atherosclerosis Atherosclerosis 2012 224 1 58 65 10.1016/j.atherosclerosis.2012.06.063 22789514

[B25] Ammirati E Monaco C Norata GD Antigen-dependent and antigen-independent pathways modulate CD4 + CD28 null T-cells during atherosclerosis Circ Res 2012 111 2 e48 e51 10.1161/CIRCRESAHA.112.271627 22773429

[B26] Gotsman I Sharpe AH Lichtman AH T-cell costimulation and coinhibition in atherosclerosis Circ Res 2008 103 11 1220 1231 10.1161/CIRCRESAHA.108.182428 19028921 PMC2662382

[B27] El CH Hassoun PM Immune and inflammatory mechanisms in pulmonary arterial hypertension Prog Cardiovasc Dis 2012 55 2 218 228 10.1016/j.pcad.2012.07.006 23009917 PMC3459180

[B28] Morillas P de Andrade H Castillo J Quiles J Bertomeu-Gonzalez V Cordero A Tarazon E Rosello E Portoles M Rivera M Bertomeu-Martínez V Inflammation and apoptosis in hypertension. Relevance of the extent of target organ damage Rev Esp Cardiol 2012 65 9 819 825 10.1016/j.recesp.2012.03.020 22771083

[B29] Hernandez-Presa MA Bustos C Ortego M Tunon J Ortega L Egido J ACE inhibitor quinapril reduces the arterial expression of NF-kappaB-dependent proinflammatory factors but not of collagen I in a rabbit model of atherosclerosis Am J Pathol 1998 153 6 1825 1837 10.1016/S0002-9440(10)65697-0 9846973 PMC1866315

[B30] Hoch NE Guzik TJ Chen W Deans T Maalouf SA Gratze P Weyand C Harrison DG Regulation of T-cell function by endogenously produced angiotensin II Am J Physiol Regul Integr Comp Physiol 2009 296 2 R208 R216 19073907 10.1152/ajpregu.90521.2008 PMC2643984

[B31] Nataraj C Oliverio MI Mannon RB Mannon PJ Audoly LP Amuchastegui CS Ruiz P Smithies O Coffman TM Angiotensin II regulates cellular immune responses through a calcineurin-dependent pathway J Clin Invest 1999 104 12 1693 1701 10.1172/JCI7451 10606623 PMC409880

[B32] Guzik TJ Hoch NE Brown KA McCann LA Rahman A Dikalov S Goronzy J Weyand C Harrison DG Role of the T cell in the genesis of angiotensin II induced hypertension and vascular dysfunction J Exp Med 2007 204 10 2449 2460 10.1084/jem.20070657 17875676 PMC2118469

[B33] Korpisalo P Karvinen H Rissanen TT Kilpijoki J Marjomaki V Baluk P McDonald DM Cao Y Eriksson U Alitalo K Ylä-Herttuala S Vascular endothelial growth factor-A and platelet-derived growth factor-B combination gene therapy prolongs angiogenic effects via recruitment of interstitial mononuclear cells and paracrine effects rather than improved pericyte coverage of angiogenic vessels Circ Res 2008 103 10 1092 1099 10.1161/CIRCRESAHA.108.182287 18832750 PMC5693290

[B34] Yamamoto S Fukumoto E Yoshizaki K Iwamoto T Yamada A Tanaka K Suzuki H Aizawa S Arakaki M Yuasa K Oka K Chai Y Nonaka K Fukumoto S Platelet-derived growth factor receptor regulates salivary gland morphogenesis via fibroblast growth factor expression J Biol Chem 2008 283 34 23139 23149 10.1074/jbc.M710308200 18559345

[B35] Cagnin S Biscuola M Patuzzo C Trabetti E Pasquali A Laveder P Faggian G Iafrancesco M Mazzucco A Pignatti PF Lanfranchi G Reconstruction and functional analysis of altered molecular pathways in human atherosclerotic arteries BMC Genomics 2009 10 13 10.1186/1471-2164-10-13 19134193 PMC2654039

[B36] Cha BY Shi WL Yonezawa T Teruya T Nagai K Woo JT An inhibitory effect of chrysoeriol on platelet-derived growth factor (PDGF)-induced proliferation and PDGF receptor signaling in human aortic smooth muscle cells J Pharmacol Sci 2009 110 1 105 110 10.1254/jphs.08282FP 19423953

[B37] Li D Ma S Yang Y Yang D Li G Zhang X Zhu J Sun M Tang B BTEB2 knockdown suppresses neointimal hyperplasia in a rat artery balloon injury model Mol Med Report 2011 4 3 413 417 21468585 10.3892/mmr.2011.438

[B38] Shim AH Liu H Focia PJ Chen X Lin PC He X Structures of a platelet-derived growth factor/propeptide complex and a platelet-derived growth factor/receptor complex Proc Natl Acad Sci U S A 2010 107 25 11307 11312 10.1073/pnas.1000806107 20534510 PMC2895058

[B39] Kim TJ Lee JH Lee JJ Yu JY Hwang BY Ye SK Shujuan L Gao L Pyo MY Yun YP Corynoxeine isolated from the hook of Uncaria rhynchophylla inhibits rat aortic vascular smooth muscle cell proliferation through the blocking of extracellular signal regulated kinase 1/2 phosphorylation Biol Pharm Bull 2008 31 11 2073 2078 10.1248/bpb.31.2073 18981576

[B40] Chintalgattu V Ai D Langley RR Zhang J Bankson JA Shih TL Reddy AK Coombes KR Daher IN Pati S Patel SS Pocius JS Taffet GE Buja LM Entman ML Khakoo AY Cardiomyocyte PDGFR-beta signaling is an essential component of the mouse cardiac response to load-induced stress J Clin Invest 2010 120 2 472 484 10.1172/JCI39434 20071776 PMC2810076

[B41] Hao L Du M Lopez-Campistrous A Fernandez-Patron C Agonist-induced activation of matrix metalloproteinase-7 promotes vasoconstriction through the epidermal growth factor-receptor pathway Circ Res 2004 94 1 68 76 10.1161/01.RES.0000109413.57726.91 14656925

[B42] Wang X Huong SM Chiu ML Raab-Traub N Huang ES Epidermal growth factor receptor is a cellular receptor for human cytomegalovirus Nature 2003 424 6947 456 461 10.1038/nature01818 12879076

[B43] Lautrette A Li S Alili R Sunnarborg SW Burtin M Lee DC Friedlander G Terzi F Angiotensin II and EGF receptor cross-talk in chronic kidney diseases: a new therapeutic approach Nat Med 2005 11 8 867 874 10.1038/nm1275 16041383

[B44] Baliga RR Pimental DR Zhao YY Simmons WW Marchionni MA Sawyer DB Kelly RA NRG-1-induced cardiomyocyte hypertrophy. Role of PI-3-kinase, p70(S6K), and MEK-MAPK-RSK Am J Physiol 1999 277 5 Pt 2 H2026 H2037 10564160 10.1152/ajpheart.1999.277.5.H2026

[B45] Nieder C Andratschke N Jeremic B Molls M Comparison of serum growth factors and tumor markers as prognostic factors for survival in non-small cell lung cancer Anticancer Res 2003 23 6D 5117 5123 14981976

[B46] Taniyama Y Morishita R Aoki M Hiraoka K Yamasaki K Hashiya N Matsumoto K Nakamura T Kaneda Y Ogihara T Angiogenesis and antifibrotic action by hepatocyte growth factor in cardiomyopathy Hypertension 2002 40 1 47 53 10.1161/01.HYP.0000020755.56955.BF 12105137

[B47] Zhu Y Hojo Y Ikeda U Shimada K Production of hepatocyte growth factor during acute myocardial infarction Heart 2000 83 4 450 455 10.1136/heart.83.4.450 10722550 PMC1729356

[B48] Morishita R Nakamura S Nakamura Y Aoki M Moriguchi A Kida I Yo Y Matsumoto K Nakamura T Higaki J Ogihara T Potential role of an endothelium-specific growth factor, hepatocyte growth factor, on endothelial damage in diabetes Diabetes 1997 46 1 138 142 10.2337/diabetes.46.1.138 8971094

[B49] Park JK Mervaala EM Muller DN Menne J Fiebeler A Luft FC Haller H Rosuvastatin protects against angiotensin II-induced renal injury in a dose-dependent fashion J Hypertens 2009 27 3 599 605 10.1097/HJH.0b013e32831ef369 19262227

[B50] Edgar R Domrachev M Lash AE Gene expression omnibus: NCBI gene expression and hybridization array data repository Nucleic Acids Res 2002 30 1 207 210 10.1093/nar/30.1.207 11752295 PMC99122

[B51] Tusher VG Tibshirani R Chu G Significance analysis of microarrays applied to the ionizing radiation response Proc Natl Acad Sci U S A 2001 98 9 5116 5121 10.1073/pnas.091062498 11309499 PMC33173

[B52] Mestres J Knegtel RMA Similarity versus docking in 3D virtual screening Perspect Drug Des Discov 2000 20 191 207 10.1023/A:1008789224614

[B53] Wu G Robertson DH Brooks CR Vieth M Detailed analysis of grid-based molecular docking: a case study of CDOCKER-A CHARMm-based MD docking algorithm J Comput Chem 2003 24 13 1549 1562 10.1002/jcc.10306 12925999

